# Perfectionism, Self-Image Goals and Compassionate Goals in Health and Mental Health: A Longitudinal Analysis

**DOI:** 10.1177/07342829241239997

**Published:** 2024-03-19

**Authors:** Taryn Nepon, Gordon L. Flett, Paul L. Hewitt

**Affiliations:** 1 7991 York University, Toronto, ON, Canada; 2 8166 University of British Columbia, Vancouver, BC, Canada

**Keywords:** perfectionism, perfectionistic self-presentation, self-image goals, compassionate goals, health

## Abstract

This research focuses on ego-focused self-image goals as central to understanding the vulnerability inherent in perfectionism and the link that perfectionism has with poorer health and emotional well-being. The present study expands theory and research on perfectionism from a unique motivational perspective through a longitudinal investigation of perfectionism, the pursuit of self-image goals related to self-improvement, and mental and physical health among 187 university students. Our central finding was that trait and self-presentational perfectionism were associated longitudinally with self-image goals and poorer mental and physical health. Longitudinal analyses showed that perfectionistic self-presentation predicted subsequent self-image goals, controlling for initial self-image goals. Additionally, self-image goals were associated with worse mental and physical health and greater loneliness and social anxiety. Collectively, our results illustrate the benefits of assessing problematic personal goals in perfectionism and the need to revise existing motivational accounts by recognizing the important role ego-involved goals play in guiding much of what perfectionists do and how they act in their daily lives.

## Introduction

Perfectionism continues to be a central topic in the psychological literature, perhaps because it is highly relevant to people’s lives. Indeed, Flett and Hewitt (2020) couched perfectionism in terms of ‘problems in living’ and this is supported by considerable research and theory over the past three decades showing that dimensions of perfectionism are associated with depression, social anxiety, mortality and suicide (Flett & Hewitt, 2024; Fry & Debats, 2009; Hewitt & Flett, 1991; Nepon et al., 2011; Smith et al., 2018). Interest in perfectionism is arguably at its peak following meta-analytic evidence indicating that levels of various trait measures of perfectionism are on the rise among younger people (Curran & Hill, 2019) and this may be fuelling the widespread prevalence of mental health problems.

One key to understanding perfectionism is to gain a better appreciation of its motivational roots. Unfortunately, although extensive research has been conducted, research on perfectionism from a motivational perspective is limited in scope and has focused narrowly on certain topics. More research is needed because fully understanding the goals and motives of perfectionists is essential. The current research focuses on one relatively neglected topic – the extent to which perfectionism relates to self-image goals involving the desire to create and defend positive self-images. To our knowledge, the only research conducted thus far was a cross-sectional investigation of the links between dimensions of perfectionism and self-image goals across four samples (Nepon et al., 2016). This work confirmed that attempts to understand perfectionism should include an emphasis on ego-involved self-image goals. Perhaps a preoccupation with self-image goals underscores the approach-avoidance conflict known to dominate perfectionists in childhood and adolescence (see Flett & Hewitt, 2022).

In the current research, we assessed both self-image and compassionate goals, and perfectionism was assessed complexly in terms of the need to be perfect (i.e. trait perfectionism) and the need to appear perfect (i.e. perfectionistic self-presentation). Trait and self-presentational perfectionism are part of the extended comprehensive model of perfectionistic behaviour (Hewitt et al., 2017). Trait perfectionism was evaluated in the current research in terms of three dimensions: self-oriented, other-oriented and socially prescribed perfectionism (Hewitt & Flett, 1991). Whereas self-oriented perfectionism involves demanding perfection of the self, other-oriented perfectionism involves demanding it from others. In contrast, socially prescribed perfectionism involves the perception of being held to impossible standards imposed on the self by others. Subsequently, Hewitt and associates (2003) introduced the construct of perfectionistic self-presentation, which applies to people who feel they must seem perfect to others (i.e. perfectionistic self-promotion) and not let others see flaws (i.e. nondisplay of imperfection) or tell them about it (i.e. nondisclosure of imperfection).

Because the interpersonal elements of perfectionism have been shown to be especially problematic (e.g. Hill et al., 1997; Molnar et al., 2023; Nepon et al., 2011), our study focused on the interpersonal components of perfectionism, which were described above: socially prescribed perfectionism and perfectionistic self-presentation. Elevated levels of both interpersonal aspects of perfectionism have been associated with depression and social anxiety (Cox & Enns, 2003; Flett & Hewitt, 2014; Hewitt et al., 2003). Research has also shown that perfectionistic self-presentation can cover up substantial psychological pain (D’Agata & Holden, 2018) and predicts unique variance in key outcomes above and beyond trait perfectionism (Casale et al., 2020). Thus, there are significant potential costs to a person’s health and well-being with this hyper-responsiveness to the social pressures to be perfect and defensively trying to appear perfect. These costs include the health consequences of feeling a chronic need to live up to pressures to be perfect. It is likely that the interpersonal components of perfectionism would be linked with the pursuit of self-image goals in a longitudinal study. Therefore, the present research employed a longitudinal design to evaluate how facets of trait and self-presentational perfectionism were associated with the pursuit of self-image goals.

Several lines of investigation have sought to explain when, how and why perfectionism is broadly linked with negative consequences. As noted above, theorists have proposed that perfectionism is characterized by a strong approach-avoidance conflict that sees the excessive over-striving of driven perfectionists as motivated by fear of failure and the need to avoid the shame and embarrassment of failure (Covington, 1992). Related themes in the perfectionism and motivation literature remain to be investigated. The current work focused on the relevance of self-image goals. This investigation stems from our contention that any broad attempt to account for the motivational orientations of perfectionistic people is incomplete without an emphasis on self-image goals. Our focus on self-image goals reflects our sense that perfectionists have feelings of inferiority that promote an ego-involved self-focus that should be reflected in an emphasis on self-image goals and perhaps less emphasis on helping others and pursuing compassionate goals. This emphasis on self-image goals as reflecting an ego system is in keeping with longstanding empirical evidence showing how perfectionists are particularly at risk when they are made to feel defensive and potentially like failures on ego-relevant important tasks and challenges (Hewitt et al., 1989). Perhaps it is the emphasis on the ego-activation element of perfectionism that accounts for the irrational importance that some people place on the need to be perfect. It is the sense that perfectionism absolutely must be obtained that is most problematic according to Albert Ellis (2002). This proposed association with ego-involved self-image goals is also in keeping with evidence suggesting that needing to be perfect is a reflection of a need for validation and engaging in activities that are steeped in a preoccupation with proving oneself (Flett et al., 2014).

Research has shown that self-image goals reflect competitive zero-sum beliefs designed to maximize rewards to the self at the expense of others, which may help explain why individuals with chronic self-image goals tend to experience interpersonal conflict and loneliness (Crocker & Canevello, 2008). People with chronic self-image goals are focused on gaining approval from others for having positive qualities (Crocker & Canevello, 2008). Self-image goals have negative health and interpersonal consequences (Canevello & Crocker, 2011) and pursuit of these goals is associated with reduced self-esteem (Taniguchi, 2022). Individuals with frequent self-image goals tend to be ego-involved and are preoccupied with being evaluated by others, which is similar to those high in interpersonal perfectionism. This self-image orientation contrasts with compassionate goals, which involve the prosocial desire to help and support others. These goals have been linked adaptively with closeness with others and receiving support from others (Crocker & Canevello, 2008).

As noted above, our interest in the self-image goals of perfectionists is consistent with the notion that people who are perfectionistic tend to be psychologically insecure about their core attributes and are ego-involved and overly focused on the actual and reflected appraisals of other people. An emphasis on self-image goals is consistent with concerns among perfectionists with elevations on the social dimensions about being negatively evaluated and seen as defective or incompetent and the shame that would result from failing to meet expectations (for a discussion, see Hewitt et al., 2017).

Historically, self-image goals or themes have been a core subject among theorists seeking to understand the roots of perfectionism. Adler (1956) posited that perfectionism involves striving for superiority to overcome inferiority feelings about the self. Missildine (1963) provided relevant insights, concluding that the striving of perfectionists is perpetuated by goals reflecting the conviction that ‘I am not good enough, I must do better’ (p. 77) and this defensive goal-striving perpetuates the inability of driven perfectionists to experience satisfaction. Also, Hollender (1965) contended that ‘perfectionism is motivated by an effort to create a better self-feeling or self-image and to obtain certain responses or supplies from other people’ (p. 99).

At present, as noted above, there has been limited investigation of the proposed link between perfectionism and self-image goals using the framework developed by Jennifer Crocker and associates. Initially, Nepon and colleagues (2016) sought to establish links between perfectionism and self-image goals in various domains. They found across three cross-sectional studies with university students that dimensions of trait and self-presentational perfectionism were linked with self-image goals in the areas of academics, friendships and self-improvement. Additional results linked trait perfectionism and perfectionistic self-presentation with rumination about self-image goals. These findings suggest some perfectionistic students are worried about image-related issues and are cognitively preoccupied with goals reflecting their self-images. Another key finding was that self-image goals mediated the links that interpersonal perfectionism had with depression and student burnout (Nepon et al., 2016). It was important to replicate these results in a longitudinal study that would also extend the prior findings. It is quite plausible that self-image goals account, in part, for why people high in interpersonal perfectionism experience social anxiety, depression, loneliness and health problems over time.

It is important to note here a major distinction between self-image goals and dimensions of perfectionism, which can be understood from the perspective of what Cantor (1990) stated about ‘having versus doing’. Cantor (1990) adopted the classic distinction in the personality field between having versus doing introduced by Gordon Allport (1937). In the current context, the focus on ‘doing’ refers to the active pursuit of self-image and compassionate goals because these goals are believed to be reflected in ongoing daily behaviours and interaction patterns. At present, much has been established in the published literature about ‘what perfectionists have’ regarding their characteristics and features, but much less is known about ‘what perfectionists actually do’ in their daily lives. The current work reflects the premise that much of what perfectionistic people actually do is geared toward achieving self-image goals.

The current research sought to expand what is known about perfectionism and self-image goals in several respects. First, perfectionism and self-image goals were uniquely examined from a longitudinal perspective. It is important to establish that perfectionism is associated with self-image goals both concurrently and longitudinally to show there is a sustained association. Another unique aspect of the present study was that we examined whether self-image goals in the area of self-improvement is a mechanism through which interpersonal perfectionism predicted worse physical and mental health over time.

The importance of focusing on perfectionistic self-presentation is suggested by conceptual analyses and empirical results. Feminist scholars highlighted the importance of striving for a perfect self-image. For instance, Bruch (1988) posited that perfectionism is largely an external façade designed to hide a negative self-identity and there is growing evidence of the negative self underscoring a need to project an image of perfection (Casale et al., 2016; Chen et al., 2015; Hewitt et al., 2003). If so, then this too suggests a plausible link with self-image goals. Longitudinal research conducted by Brown and Gilligan (1992, 1993) with adolescent girls showed that over time they would suppress their true emotions due, at least in part, to the pressure of living up to prescribed expectations and a perfect self-image. Collectively, these results seem to point to the existence of people who define themselves and are more invested in seeming perfect rather than actually being perfect.

Given these observations, the present research employed a longitudinal design to evaluate how trait and self-presentational perfectionism were associated with the pursuit of self-image goals in the area of self-improvement. Self-improvement should be especially salient among extreme perfectionists who are preoccupied with addressing their imperfections to enhance the self. Qualitative research among 20 highly perfectionistic university students found that striving for self-improvement was a salient and common theme that emerged in life narrative interviews (Farmer et al., 2017). The current research focused on what perfectionists actually do in terms of their ego-involved goal pursuits by evaluating the main premise that perfectionism is linked with self-image goals for self-improvement. The current research also assessed compassionate goals and incorporated a longitudinal perspective. Most notably, we sought to determine whether perfectionism would be associated over time with the pursuit of self-image goals and whether this would be apparent for both trait and self-presentational perfectionism. Our longitudinal focus reflects, in part, the need to evaluate the extent to which the proposed association between perfectionism and self-image goals reflects a stable tendency.

Although there has been no longitudinal research to date exploring the associations between perfectionism and self-image goals, it has been well-established in longitudinal research that perfectionism predicts distress over time. For instance, socially prescribed perfectionism predicted depression over time among adolescents (O’Connor et al., 2010). Other longitudinal research found links between perfectionism and poor physical health (see Molnar et al., 2018, for a review). Unidimensional perfectionism predicted worse symptoms of physical health over time in university students (Pritchard et al., 2007). Fry and Debats (2009) explored the longer-term health consequences of trait perfectionism. They evaluated middle-aged Canadians in a seven-year study and found that self-oriented and socially prescribed perfectionism predicted all-cause early mortality. To our knowledge, there has been no longitudinal research and limited cross-sectional research on the links between perfectionism and loneliness. However, existing research does support the link between perfectionism and loneliness (e.g. Flett et al., 1996).

Additionally, there has been longitudinal research on self-image and compassionate goals and this research points to the vulnerability inherent in the pursuit of self-image goals. One such study revealed that self-image goals predicted higher distress over time in university students, while compassionate goals predicted lower distress over time (Crocker et al., 2010). Given that interpersonal perfectionism and self-image goals are both linked with distress, it stands to reason that those high in interpersonal perfectionism and self-image goals will likely be at risk of experiencing poor health outcomes.

### Hypotheses in the Current Study

We hypothesized in the present study that the trait and self-presentational components of perfectionism would be positively correlated with self-image goals, and this would be found both cross-sectionally and longitudinally. It was also expected that trait and self-presentational perfectionism at Time 1 would predict unique variance in self-image goals at Time 2, over and above trait perfectionism and self-image goals at Time 1. We further hypothesized that interpersonal perfectionism (i.e. socially prescribed perfectionism and perfectionistic self-presentation) would be associated longitudinally with depression, social anxiety, loneliness, and poor physical and mental health. Lastly, we hypothesized that Time 2 self-image goals would mediate the links between Time 1 interpersonal perfectionism and Time 2 physical and mental health. One unique contribution of this study was that self-image goals for self-improvement was the specific mechanism through which interpersonal perfectionism was possibly associated with physical and mental health over time. No explicit hypotheses were made concerning compassionate goals because this element of our investigation had an exploratory focus.

## Method

### Participants

Our sample comprised 187 university students (135 women, 52 men), with a mean age at Time 1 of 19.7 years (*SD* = 3.4). Participants were recruited through an undergraduate research participant pool. They received credit towards their final introductory psychology grades in exchange for their participation following each time point for a total of two credits. Most participants (59.4%) were in their first year of study. The most commonly reported intended major was psychology (29.9%).

### Procedure

Participants completed self-report questionnaires over the Internet at two time points: the first term in November and the second term from February to March. Participants first provided their informed consent. This research was reviewed and approved for compliance to research ethics protocols at a large Canadian university.

### Measures

Most measures were assessed at both time points, except for the Multidimensional Perfectionism Scale (MPS) and the Perfectionistic Self-Presentation Scale (PSPS). These measures were assessed only at Time 1.

#### Multidimensional Perfectionism Scale

This 45-item scale measures three dimensions of trait perfectionism: self-oriented perfectionism (e.g. ‘When I am working on something, I cannot relax until it is perfect’); other-oriented perfectionism (e.g. ‘Everything that others do much be of top-notch quality’); and socially prescribed perfectionism (e.g. ‘The better I do, the better I am expected to do’). Considerable research has demonstrated that this scale is multidimensional with good reliability and validity in student and clinical samples (Hewitt & Flett, 1991, 2004).

#### Perfectionistic Self-Presentation Scale

This 27-item inventory has three facets: perfectionistic self-promotion (e.g. ‘I must always appear to be perfect’); nondisplay of imperfection (e.g. ‘Errors are much worse if they are made in public rather than in private’); and nondisclosure of imperfection (e.g. ‘I should solve my own problems rather than admit them to others’). The PSPS possesses good psychometric properties (Hewitt et al., 2003).

#### Compassionate and Self-Image Goals Scale

This 18-item scale measures self-image goals (e.g. ‘get others to recognize or acknowledge your positive qualities’) and compassionate goals (e.g. ‘be supportive of others’) in the area of self-improvement goals. Before rating the items with respect to the previous week, these instructions were provided:

Please reflect for a moment on your self-improvement goals in general (e.g., exercise more, eat healthier, be a better person overall). While keeping these goals in mind, answer each of the following statements using the scale shown below.

We added four self-image goal items to increase scale content related to approach goals (i.e. ‘let others see that you are capable’, ‘do things to try to earn the approval of others’, ‘display your strengths’ and ‘do things to establish your worth to others’). These additional items were used in previous research (Nepon et al., 2016). Both subscales have good psychometric properties (Crocker & Canevello, 2008). The additional items possess good reliability (Nepon et al., 2016).

#### Center for Epidemiological Studies-Depression Scale

This 20-item questionnaire assesses the frequency of depressive symptoms over the last week (e.g. ‘I was bothered by things that usually don’t bother me’). The Center for Epidemiological Studies-Depression Scale (CES-D) has sufficient psychometric properties (Radloff, 1977).

#### Liebowitz Social Anxiety Scale

This 24-item scale has two subscales addressing social interaction (e.g. ‘Expressing a disagreement or disapproval to people you don’t know very well’) and performance (e.g. ‘Acting, performing or giving a talk in front of an audience’) situations. Respondents rate how frequently they felt fear or anxiety and how frequently they avoided each activity over the past week. The psychometric properties of the Liebowitz Social Anxiety Scale (LSAS) are well-established (Liebowitz, 1987).

#### UCLA Loneliness Scale

This 20-item scale measures perceived loneliness. Sample items include ‘How often do you feel alone’? and ‘How often do you feel isolated from others’? This scale possesses good reliability and validity (Russell et al., 1980).

#### SF-36v1 Health Survey

Four subscales of the SF-36v1 assessed self-reported physical health symptoms over the last four weeks. A summary score was used based on these four scales. The physical functioning subscale comprises 10 activities respondents may do on a typical day and are asked if their health limits them in these activities (e.g. ‘Climbing one flight of stairs’). The role-physical subscale comprises four items assessing if any problems were experienced with work or regular activities as a result of physical health (e.g. ‘cut down the amount of time you spent on work or other activities’?). The bodily pain subscale comprises two items, with one measuring pain severity and the second measuring the extent to which pain interferes with daily functioning. The general health subscale has six items. The first item asks respondents to rate their perceived health and the second asks respondents to rate their general health now compared to one year ago. Another sample item is ‘My health is excellent’.

Four subscales assessed mental health problems over the past four weeks and form a composite mental health measure. They assess vitality (e.g., “Did you feel full of pep?”), social functioning (i.e., whether their health interfered with normal social activities), role-emotional functioning (i.e., whether problems were experienced with work or regular daily activities as a result of any emotional problems), and mental health (i.e., “Have you felt calm and peaceful?”)

We focused on the two summary scores, with higher scores indicating better physical and mental health. The psychometric properties of the SF-36v1 have been well-established (Ware et al., 1993). The two summary scores possess good reliability and validity (Ware et al., 1994).

## Results

### Correlational Analyses

Our initial correlations focused on the associations among the Time 1 measures. As expected, Time 1 self-image goals were associated with Time 1 trait perfectionism and perfectionistic self-presentation, but there were no links between compassionate goals and perfectionism. Self-image goals were associated with self-oriented and socially prescribed perfectionism (respective *r*’s of .21 and .32) and all three PSPS facets (*r*’s ranging from .34 to .40). In almost all instances, socially prescribed perfectionism and all PSPS facets were significant correlates across the measures of health, mental health, loneliness and social anxiety. For example, at Time 1, the mental health composite was associated with all four perfectionism measures (*r*’s ranging from −.22 to −.42) and loneliness (*r*’s ranging from .32 to .53). The exception was Time 1 physical health. Poorer physical health at Time 1 was associated with socially prescribed perfectionism (*r* = −.34), nondisplay of imperfection (*r* = −.26) and nondisclosure of imperfection (*r* = −.38).

Table 1 displays the correlations that Time 1 perfectionism dimensions and Time 1 self-image and compassionate goals have with the Time 2 variables. Table 1 also shows there were moderate correlations over time between Time 1 and Time 2 self-image goals and Time 1 and Time 2 compassionate goals.

**Table 1.** Correlations Between Time 1 Perfectionism and Goals, and Time 2 Goals, Depression, Social Anxiety, Loneliness, and Physical and Mental Health.

	Time 2						
	Image	Comp	CES-D	LSAS	UCLA	Physical	Mental
Time 1							
SOP	.16*	.06	−.02	−.07	−.06	.13	.002
OOP	−.07	−.20**	.02	−.02	.07	.06	−.02
SPP	.22**	−.18*	.40**	.30**	.45**	−.28**	−.33**
PSP	.40**	−.10	.19**	.24**	.23**	−.06	−.18*
Ndisplay	.36**	−.04	.36**	.49**	.42**	−.17*	−.39**
Ndisclose	.25**	−.13	.40**	.33**	.44**	−.26**	−.34**
Image	.49**	.08	.18*	.18*	.22**	−.13	−.21**
Comp	.23**	.34**	−.04	.05	−.03	.15*	−.10

*Note. N* = 187. *p < .05, **p < .01, two-tailed. The abbreviations are SOP = Self-Oriented Perfectionism; OOP = Other-Oriented Perfectionism; SPP = Socially Prescribed Perfectionism; PSP = Perfectionistic Self-Promotion; Ndisplay = Nondisplay of Imperfection; Ndisclose = Nondisclosure of Imperfection; Image = Self-Image Goals for Self-Improvement; Comp = Compassionate Goals for Self-Improvement; CES-D = Center for Epidemiological Studies-Depression Scale; LSAS = Liebowitz Social Anxiety Scale; UCLA = UCLA Loneliness Scale; Physical = Physical Health; and Mental = Mental Health.

As seen in Table 1, Time 1 self-oriented, socially prescribed perfectionism and all PSPS facets were positively correlated with Time 2 self-image goals. Again, compassionate goals were not linked with perfectionism, other than Time 1 other-oriented and socially prescribed perfectionism being negatively correlated with Time 2 compassionate goals.

Time 1 socially prescribed perfectionism and all PSPS facets were positively correlated with Time 2 depression, social anxiety and loneliness. Time 1 socially prescribed perfectionism, nondisplay of imperfection and nondisclosure of imperfection were associated with poorer physical and mental health at Time 2; perfectionistic self-promotion at Time 1 was associated with worse mental health at Time 2, but not associated with physical health at Time 2. Additionally, self-image and compassionate goals were positively correlated with each other at Time 1 (*r* = .50, *p* < .01) and Time 2 (*r* = .36, *p* < .01).

Regarding the links that Time 1 self-image and compassionate goals had with the Time 2 outcomes, Time 1 self-image goals were correlated with greater depression, social anxiety, loneliness and worse mental health at Time 2, while Time 1 compassionate goals were only correlated with better physical health at Time 2 (see Table 1).

Regarding the cross-sectional correlations that self-image and compassionate goals had with the outcomes at both time points, Time 1 self-image goals were correlated with Time 1 depression (*r* = .25, *p* < .01), social anxiety (*r* = .21, *p* < .01), loneliness (*r* = .21, *p* < .01) and negatively correlated with physical health (*r* = −.15, *p* < .05) and mental health (*r* = −.19, *p* < .01). Time 1 compassionate goals were not associated with the Time 1 outcomes. Time 2 self-image goals were correlated with Time 2 depression (*r* = .21, *p* < .01), social anxiety (*r* = .21, *p* < .01), loneliness (*r* = .20, *p* < .01) and negatively correlated with mental health (*r* = −.16, *p* < .05). In contrast, Time 2 compassionate goals seemed adaptive with small associations with Time 2 depression (*r* = −.15, *p* < .05), loneliness (*r* = −.23, *p* < .01) and better physical health (*r* = .16, *p* < .05).

#### Partial Correlations

Partial correlations determined whether the established links between Time 1 perfectionism and Time 2 self-image goals remained significant after controlling for Time 2 compassionate goals. These analyses seemed essential given the positive correlation between self-image and compassionate goals. The correlations remained significant after controlling for compassionate goals. Specifically, Time 1 self-oriented, socially prescribed perfectionism and all PSPS facets were still significantly linked with Time 2 self-image goals, with partial correlations ranging from .15 to .47.

Partial correlations also explored whether the links between Time 1 perfectionism dimensions and Time 2 compassionate goals remained significant after controlling for Time 2 self-image goals. The negative correlations Time 1 other-oriented and socially prescribed perfectionism had with Time 2 compassionate goals were still significant after controlling for Time 2 self-image goals. Several additional negative correlations with Time 2 compassionate goals emerged after controlling for Time 2 self-image goals. All Time 1 PSPS facets were negatively linked with Time 2 compassionate goals, after controlling for Time 2 self-image goals, with partial correlations from −.18 to −.28.

### Regression Analyses

A hierarchical multiple regression analysis evaluated whether Time 1 perfectionistic self-presentation predicted Time 2 self-image goals, above and beyond Time 1 trait perfectionism. First, we screened for normality and the distribution did not differ significantly from normal. This analysis controlled for initial levels of self-image goals (i.e. at Time 1). This regression was conducted with Time 1 self-image goals entered into the first predictor block, Time 1 MPS dimensions entered into the second predictor block, followed by Time 1 PSPS facets, and with Time 2 self-image goals for self-improvement as the outcome (see Table 2). Time 1 trait perfectionism dimensions did not significantly predict variance in Time 2 self-image goals, after controlling for Time 1 self-image goals. However, the block with the Time 1 PSPS subscales did significantly predict an additional 5.6% of the variance in Time 2 self-image goals, *F*(7, 179) = 11.98, *p* < .001. Regarding individual predictors, Time 1 perfectionistic self-promotion predicted increased Time 2 self-image goals. An unexpected finding was that Time 1 other-oriented perfectionism was negatively associated with Time 2 self-image goals.

**Table 2.** Summary of Hierarchical Multiple Regression for Time 1 Variables Predicting Time 2 Self-Image Goals.

Variable	B	SE B	ß
Step 1			
Time 1 self-image goals	.46	.06	.49***
Step 2			
Self-oriented perfectionism	.004	.004	.08
Other-oriented perfectionism	−.01	.005	−.14*
Socially prescribed perfectionism	.004	.004	.07
Step 3			
Perfectionistic self-promotion	.02	.006	.27**
Nondisplay of imperfection	.006	.005	.11
Nondisclosure of imperfection	−.007	.008	−.08

*Note. R*^2^ = .240 for Step 1; ∆*R*^2^ = .023 for Step 2; ∆*R*^2^ = .056 for Step 3. *p < .05, **p < .01, ***p < .001.

Several hierarchical multiple regression analyses were performed to explore whether Time 1 trait and self-presentational perfectionism predicted unique variance in the Time 2 outcomes, after controlling for initial levels of the outcomes. First, we screened for normality; other than loneliness, all distributions differed significantly from normal. Thus, the robust bootstrapping procedure was used for most regressions because it does not impose the normality assumption. Only the significant findings are reported. The MPS and PSPS dimensions predicted additional variance in Time 2 social anxiety; however, there were no significant individual predictors. The perfectionism dimensions also predicted additional variance in loneliness, and Time 1 socially prescribed perfectionism predicted increased loneliness at Time 2. Lastly, perfectionism predicted additional variance in Time 2 physical health. Regarding individual predictors, Time 1 socially prescribed perfectionism predicted worse physical health at Time 2, but Time 1 self-oriented perfectionism predicted better physical health at Time 2. Specific details are available upon request.

### Mediation Analyses

Two structural equation models were tested to evaluate whether self-image goals would mediate the links between Time 1 interpersonal perfectionism (i.e. socially prescribed perfectionism and perfectionistic self-presentation) and the various outcomes at Time 2 (i.e. depression, social anxiety, loneliness, and the mental and physical health summary scores). The two models differed in terms of the outcomes: the first model included a latent distress factor comprising depression, social anxiety, and loneliness, and the second model included the physical health and mental health summary scores. Initially, one model was tested; however, the separate models were both better fits than the initial model. These models were evaluated to determine whether it was possible to replicate and expand upon the previous cross-sectional findings (Nepon et al., 2016) by showing self-image goals as a key mediator of the longitudinal links between interpersonal perfectionism and both mental and physical health.

#### Model 1

**Figure 1.**
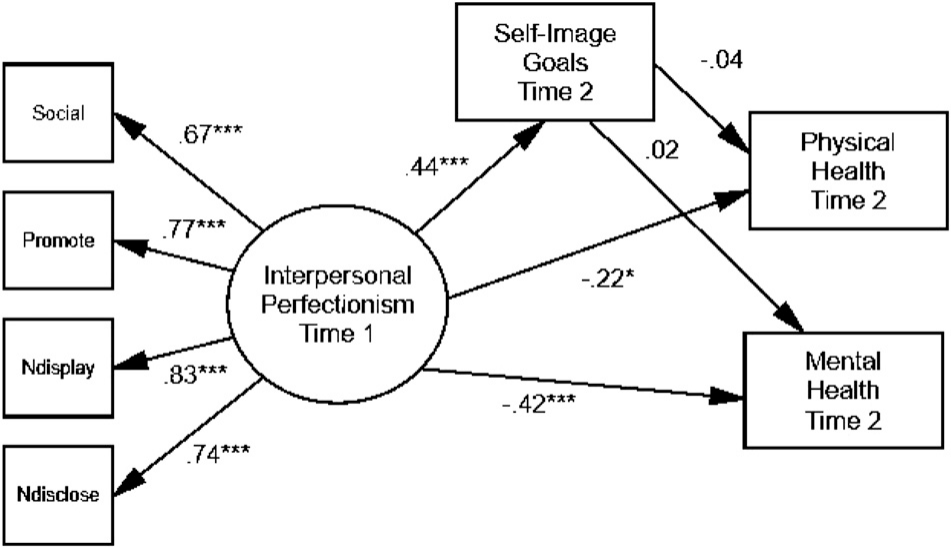
Final model of Time 1 interpersonal perfectionism, Time 2 self-image goals, and Time 2 physical and mental health. *Note.* ***p < .001. Standardized parameter estimates are shown. Error terms and the correlation between physical and mental health have been omitted for ease of presentation. The abbreviations are Social = Socially Prescribed Perfectionism; Promote = Perfectionistic Self-Promotion; Ndisplay = Nondisplay of Imperfection; and Ndisclose = Nondisclosure of Imperfection.

The first hypothesized model was tested using maximum likelihood estimation procedures (see Figure 1). The predictor was the interpersonal perfectionism latent factor at Time 1, the mediator was self-image goals at Time 2, and the outcomes were the mental and physical health summary scores at Time 2. The correlation between the two summary scores was taken into account. This model was an adequate fit, *χ*^2^ (11) = 33.69, *p* = .000, CFI = .95, TLI = .90, SRMR = .05, RMSEA = .11, 90% CI [.07, .15], *p*_close_ = .013. Time 1 interpersonal perfectionism was positively associated with Time 2 self-image goals. However, self-image goals were not significantly related to either outcome. The mediator would need to be significantly linked with the outcomes for mediation to be significant. This model does demonstrate that Time 1 interpersonal perfectionism was significantly and negatively linked with Time 2 mental and physical health when Time 2 self-image goals were included in the model. Therefore, interpersonal perfectionism was associated with worse mental and physical health three months later. Physical and mental health were correlated with each other (*r* = .50, *p* < .001).

#### Model 2

**Figure 2.**
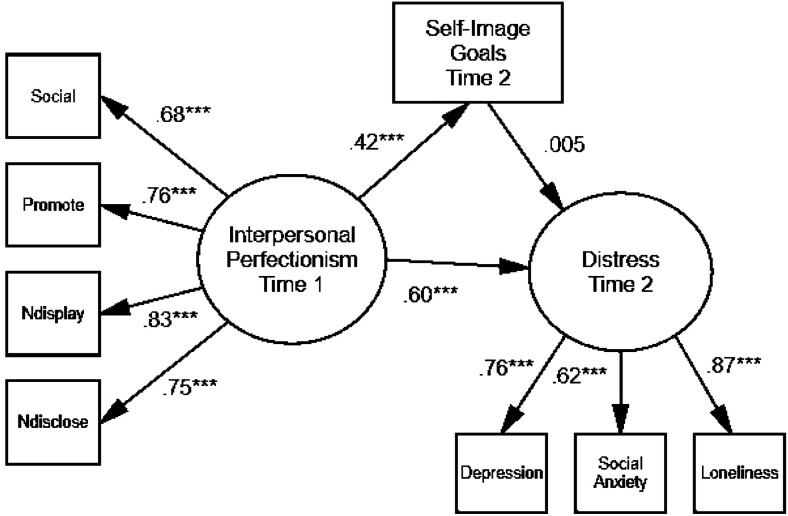
Final model of Time 1 interpersonal perfectionism, Time 2 self-image goals, and Time 2 distress. *Note.* ***p < .001. Standardized parameter estimates are shown. Error terms have been omitted. The abbreviations are Social = Socially Prescribed Perfectionism; Promote = Perfectionistic Self-Promotion; Ndisplay = Nondisplay of Imperfection; and Ndisclose = Nondisclosure of Imperfection.

Next, the second hypothesized model was evaluated (see Figure 2). The predictor was the same interpersonal perfectionism latent factor at Time 1, the mediator was self-image goals at Time 2, and the outcome was a distress latent factor comprising Time 2 depression, social anxiety and loneliness. This model was an adequate fit, χ. (18) = 57.69, *p* = .000, CFI = .93, TLI = .89, SRMR = .06, RMSEA = .11, 90% CI [.08, .14], *p*close = .001. Time 1 interpersonal perfectionism was positively associated with Time 2 self-image goals. However, self-image goals were not significantly related to the distress factor. Because this link between the mediator and the outcome was not significant, mediation was not found. This model does show that Time 1 interpersonal perfectionism was significantly and positively linked with Time 2 distress when Time 2 self-image goals were included in the model. Collectively, these models demonstrate that while mediation was not found, interpersonal perfectionism was associated with greater self-image goals, and worse physical and mental health over time.

## Discussion

The present longitudinal study uniquely examined the relations among perfectionism, goals in the area of self-improvement and various outcome indicators. Our indices included depression, social anxiety, loneliness, and physical and mental health problems. This research focused on whether the links established in cross-sectional research by Nepon et al. (2016) between perfectionism and self-image goals would be evident in longitudinal research with an emphasis on self-improvement goals.

As expected, the association between perfectionism and self-image goals was confirmed in the present study. In addition, the results confirmed that perfectionism longitudinally predicts the pursuit of self-image goals. Correlational analyses linked initial trait perfectionism and self-presentational perfectionism with subsequent self-image goals and it was further established that there is temporal stability in the pursuit of self-image goals and compassionate goals with the test-retest reliability being stronger for self-image goals. Our analyses indicated further that Time 1 perfectionistic self-presentation uniquely predicted Time 2 self-image goals, over and above trait perfectionism and controlling for Time 1 self-image goals. Collectively, these results further attest to the link between perfectionistic self-presentation and self-image goals. It is important to consider these results in light of the crucial distinction Cantor (1990) had made between ‘having versus doing’, which was described earlier. In the present study, we demonstrated that individuals with an enduring desire to look perfect have the tendency to pursue self-image goals related to their quest for self-improvement.

Interpersonal facets of perfectionism and self-image goals were also associated with various indicators of physical and mental health and issues in psychosocial adaptation (i.e. loneliness and social anxiety). However, analyses indicated that our evidence did not support self-image goals as a mediator of the association between perfectionism and physical and mental health. Overall, the current longitudinal study extends previous research by providing further evidence for the usefulness of a motivational approach focused on self-image goals when seeking to understand perfectionists and why they are so driven. This work can be interpreted as a reminder that perfectionism reflects issues involving the self-concept and self-image. Given that the self-image goals measure tends to jointly reflect approach and avoidance tendencies, the motivational conflict for certain perfectionists extends to this form of goal striving.

Correlational analyses revealed that self-oriented, socially prescribed perfectionism and perfectionistic self-presentation were positively correlated with self-image goals for self-improvement, both concurrently and longitudinally. These findings replicated and extended previous results. Most notably, the correlations between self-image goals and perfectionistic self-presentation were more robust than the links between self-image goals and trait perfectionism. This pattern of correlations reflects our sense that perfectionistic self-presentation is a style fuelled constantly by ego-involved goals and a preoccupation with seeming successful and gaining approval and recognition and not seeming incompetent, deficient, and avoiding disapproval and other forms of negative social regard. It is also conceivable that the pursuit of self-image goals can account developmentally for which people experiencing social pressure to be perfect are most likely to respond to socially prescribed perfectionism by trying to seem perfect. Future research should explore whether the pursuit of self-image goals contributes to other individual differences involved in perfectionism (e.g. concern over mistakes and doubts about actions) and whether conducting assessments at multiple timepoints can determine if there is a reciprocal association between self-image goals and components of perfectionism.

Parenthetically, it should be noted that links were still found between self-oriented perfectionism and self-image goals in the current study, both concurrently (*r* = .21) and longitudinally (*r* = .16). However, these associations were considerably less robust than the association found between self-oriented perfectionism and self-image goals reported in Nepon et al. (2016). The correlations were typically .40 or higher in this previous work. One possibility worth exploring is that perhaps self-oriented perfectionism entails greater vulnerability when self-image goals are the focus and have been primed among people who are driven and feel they must be perfect.

Partial correlation analyses in our study confirmed that the established significant correlations between Time 1 perfectionism dimensions and Time 2 self-image goals were still evident despite controlling for Time 2 compassionate goals. Further, Time 1 socially prescribed perfectionism and most perfectionistic self-presentation facets were positively associated with Time 2 depression, social anxiety, and loneliness, and negatively associated with Time 2 physical and mental health.

Other findings are in keeping with the destructiveness of social pressures to be perfect (see Flett et al., 2022). That is, interpersonal perfectionism at Time 1 (i.e. socially prescribed perfectionism and perfectionistic self-presentation) was associated with worse physical health at Time 2 and was associated to a greater degree with worse mental health at Time 2 (see Figure 1). This research represents another illustration of the potential link between perfectionism and physical health and it is one of few investigations to do so from a longitudinal perspective. Interpersonal perfectionism also predicted subsequent adjustment in terms of a composite of depression, social anxiety and loneliness (see Figure 2). Supplementary regression analyses indicated that perfectionism predicted changes in several indices over time; in particular, Time 1 socially prescribed perfectionism predicted increased loneliness and decreased physical health. These results are consistent with predictions of the perfectionism social disconnection model (Hewitt et al., 2017). This model proposes that the interpersonal perfectionism dimensions are associated with distress due to the tendency to experience subjective and objective social disconnection. The findings with perfectionism and social anxiety provide support for a model posited by Alden and colleagues (2002) and extended by Flett and Hewitt (2014). Our findings also expanded on previous work showing links between social anxiety and trait and self-presentational perfectionism (Nepon et al., 2011).

Another unique goal of the present study was to assess whether self-image goals for self-improvement at Time 2 would mediate the associations between Time 1 interpersonal perfectionism and Time 2 physical and mental health. Two mediational models were tested and while self-image goals at Time 2 did not mediate the links that Time 1 interpersonal perfectionism had with any of the mental and physical health outcomes at Time 2, the analyses revealed that interpersonal perfectionism was associated with worse mental and physical health over time.

Our primary results focused on composite outcome measures, but individual tests showed that trait perfectionism (i.e. socially prescribed perfectionism) predicted significantly higher loneliness and worse physical health over time after taking into account initial levels of health and loneliness. These findings supplement past research, which mostly examined perfectionism and both loneliness and physical health in cross-sectional research.

While self-image goals at Time 2 were correlated concurrently with greater depression, social anxiety, loneliness, and worse mental health, compassionate goals at Time 2 were correlated concurrently with lower depression and loneliness, and better physical health. These findings are consistent with previous research showing that self-image goals are linked with worse psychological health, while compassionate goals are linked with greater psychological health (Crocker & Canevello, 2008; Crocker et al., 2010). However, longitudinal analyses found a weak association between self-image goals and poorer mental health, and an even weaker association between compassionate goals and better physical health. These findings suggest the need for further longitudinal research on self-image and compassionate goals in health given other limited evidence that self-image goals predict negative outcomes over time and compassionate goals predict positive outcomes over time (Duarte & Pinto-Gouveia, 2015). It is worth investigating in future research, perhaps with older adults, whether fewer compassionate goals are associated with health and mental health problems, especially among perfectionists with large imbalances in their personal ratios of self-image to compassionate goals because they may lack protective forms of social interest. Compassionate goals may also be adaptive in terms of the promotion of positive health behaviours for the self and others during challenging times, such as the current COVID-19 pandemic. Indeed, compassionate goals uniquely predicted more frequent COVID-19 health behaviours across several studies (Ospina et al., 2021). Compassionate goals also predicted a desire to protect oneself, close others and distant others from COVID-19, and these reasons mediated the compassionate goals-COVID-19 health behaviours link (Ospina et al., 2021).

### Limitations and Future Directions

Limitations of the current study should be noted. First, despite our longitudinal focus, causal statements are not warranted. Future experimental research should explore the causal associations, if any, among perfectionism, self-image goals and health over time. Second, all measures in our study were based on self-reports; thus, in keeping with arguments proposed by Flett and associates (2005), future research should incorporate additional measures, such as informant reports and physiological indicators of stress. However, given this focus on self-reports, we felt it was all the more important to show that the link between perfectionism and self-image goals could be detected over time. Third, the generalizability of this work should be established by re-examining the links in various different age groups and among individuals with chronic illnesses. Specifically, we may detect a goals and physical health association among older adults and it needs to be considered in people coping with chronic illness as well because frequent self-image goals and fewer compassionate goals should be quite problematic in these instances.

Another avenue for future research is to evaluate how self-image and compassionate goals for self-improvement are linked with other broad perfectionism domains, such as those assessed by the Big Three Perfectionism Scale. This inventory measures three higher-order global factors of dispositional perfectionism: rigid perfectionism, self-critical perfectionism and narcissistic perfectionism (Smith et al., 2016). While all three factors would be important to assess in terms of their associations with self-image goals, a focus on narcissistic perfectionism should prove informative. This higher-order factor comprises four facets: other-oriented perfectionism, hypercriticism, entitlement and grandiosity. The defensiveness and fragility of narcissistic perfectionists should translate into a preoccupation with self-image goals.

Additional future research could assess potential mediators of the associations between perfectionism and self-image goals to advance our understanding of the consistent links established in the present research and prior research (Nepon et al., 2016). For example, mindfulness may mediate the link between perfectionism and self-image goals. Self-image goals were negatively linked with mindfulness across three studies (Stewart et al., 2018) and various elements of the perfectionism construct predict low mindfulness (Flett et al., 2020).

In summary, the present study extended cross-sectional findings from prior research by showing there were both cross-sectional and longitudinal associations between perfectionism dimensions and self-image goals in the self-improvement domain. Perfectionistic self-presentation at Time 1 predicted unique variance in self-image goals at Time 2, over and above Time 1 trait perfectionism and self-image goals. Furthermore, interpersonal perfectionism at Time 1 was significantly linked with worse mental and physical health over time, even though self-image goals did not mediate these links. Collectively, these results illuminate the self and identity processes underscoring the vulnerabilities of perfectionistic university students.
